# From Rust to Rhythm: A Case of Toxin-Induced Junctional Bradycardia

**DOI:** 10.7759/cureus.94842

**Published:** 2025-10-18

**Authors:** Jayden Patel, Tha H Nyi, Roy Jogiya

**Affiliations:** 1 Acute Internal Medicine, Royal Free London NHS Foundation Trust, London, GBR; 2 Cardiology, Barts Health NHS Trust, London, GBR; 3 Cardiology, Kingston and Richmond NHS Foundation Trust, London, GBR

**Keywords:** cardiac arrhyrthmias, cardiac rhythm monitoring, inhalation exposure, isoprenaline infusion, junctional bradycardia

## Abstract

We present a case of a 68-year-old gentleman who developed acute symptomatic junctional bradycardia secondary to inhalation of Paint-Over-Rust 15 solution (POR-15), an anti-rust agent commonly used in both occupational and home environments. Management of this case included the use of isoprenaline infusion and close monitoring at a cardiac center for eight days. The patient made a full recovery with complete resolution of the symptoms without the need for a permanent pacemaker and further cardiology follow-up. Although rare, this case highlights the importance of considering inhalation exposure in patients presenting with arrhythmias, as this is often under-recognized in clinical practice. It also highlights the importance of appropriate cardiac monitoring and the early involvement of specialist centers. To our knowledge, this is the first reported case directly related to POR-15 solution.

## Introduction

In the UK alone, there are over 2 million people living with arrhythmias [1] with common risk factors including alcohol, smoking, hypertension, and electrolyte imbalances. Paint-Over-Rust 15 solution (POR-15) is commonly used as an anti-rust agent, and its chemical composition is predominantly made up of hydrocarbons. Two hydrocarbons, toluene and dichloromethane, are known arrhythmogenic agents. Although inhaled hydrocarbon-based chemicals, both medically and recreationally, are commonly associated with respiratory effects, their cardiac complications are rarely documented. Few case reports of hydrocarbon-based chemicals have described cardiac sequelae of chemical inhalation, including atrial fibrillation, bradycardia, cardiomyopathies, and acute heart failure [2-4]. Junctional bradycardia represents sino-atrial node failure, with the atrioventricular node providing a slower escape rhythm resulting in a higher risk of inadequate cardiac output and asystole. 

We present a case of symptomatic junctional bradycardia secondary to incidental inhalation of POR-15 solution. 

## Case presentation

**Figure 1:**
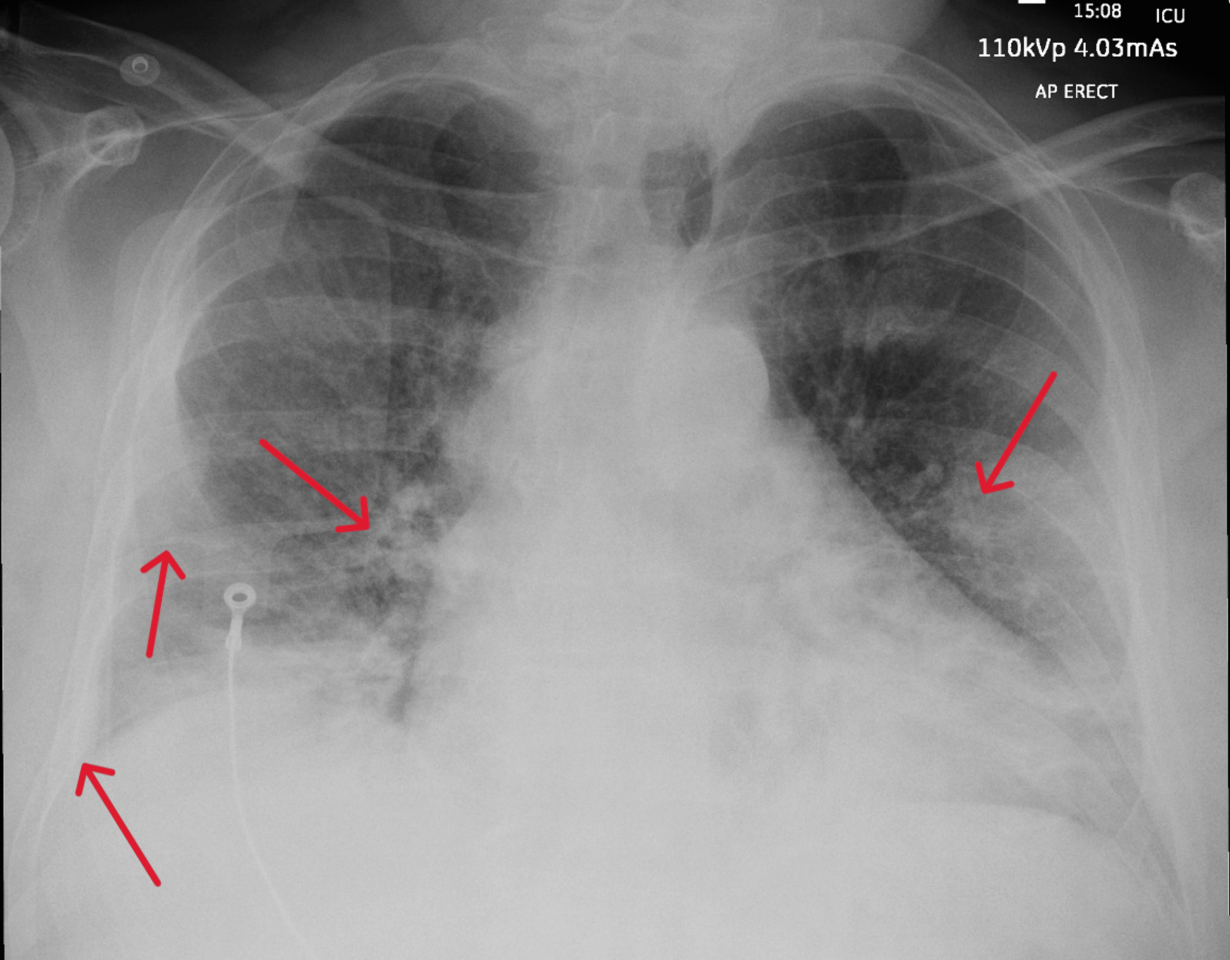
Admission chest X-ray demonstrating evidence of pulmonary congestion and infiltrates (marked with red arrows)

**Figure 2:**
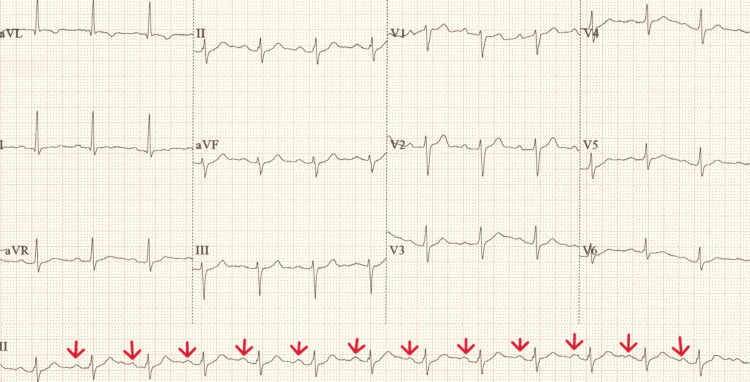
Initial ECG taken on admission demonstrating normal sinus rhythm (marked with red arrows identifying P waves before every QRS complex) with borderline prolonged PR interval and left-axis deviation ECG, electrocardiogram.

A 68-year-old man with a past medical history of obstructive sleep apnoea (OSA) for which he uses continuous positive airway pressure (CPAP) at night, hypertension and asthma presented to a district general hospital with difficulty in breathing and lightheadedness. He was otherwise fit and well. Before this presentation, he reported that he was using POR-15 solution to remove rust from his vehicle for 2 hours. Although he was wearing his usual CPAP mask as protection to avoid accidental inhalation, he acknowledged the possibility that the valve of the mask was possibly left open. On presentation, his oxygen saturation level was 97% on 15 litres via a non-rebreather mask. His blood pressure was 135/87 with a heart rate of 99 beats per minute and a respiratory rate of 20. His Glasgow Coma Score (GCS) was 15/15. The initial arterial blood gas analysis revealed a severe type 1 respiratory failure (Table 1), with his blood tests showing raised inflammatory markers and troponin (Table 2). A chest X-ray showed bilateral pulmonary infiltrates with moderate pulmonary congestion (Figure 1) whilst his electrocardiogram (ECG) showed normal sinus rhythm with left-axis deviation (Figure 2). 

**Table 1:** Arterial blood gas taken on admission showing type 1 respiratory failure pH, potential of hydrogen; pCO_2_, partial pressure of carbon dioxide; pO_2_, partial pressure of oxygen; cHCO_3_, actual bicarbonate; SBE, standard base excess; ctHb, concentration of total haemoglobin; sO_2_, oxygen saturation; fO_2_Hb, fraction of oxyhaemoglobin; fCOHb, fraction of carboxyhaemoglobin; fMetHb, fraction of methaemoglobin; cK+, potassium level in blood; cNa+, sodium level in blood; cLac, lactate level in blood; Glu, glucose level in blood; FiO_2_, fraction of inspired oxygen. Abnormal results are identified in bold.

Parameter	Result	Unit	Normal Range
pH	7.44	-	7.35-7.45
pCO_2_	4.55	kpa	4.7-6.4
pO_2_	9.16	kpa	11.0-14.4
CHCO_3_	24.5	mmol/L	22.0-29.0
SBE	-0.3	mmol/L	-2.0 to 3.0
ctHb	147	g/L	130-170
sO_2_	94.9	%	94.0-98.0
fO_2_Hb	93.4	%	-
fCOHb	0.8	%	0.0-2.9
fMetHb	0.8	%	0.0-1.4
cK+	3.7	mmol/L	3.4-4.9
cNa+	140	mmol/L	133-146
cLac	2.0	mmol/L	0.4-0.8
Glu	5.1	mmol/L	3.5-6.0
Temperature	37	Degrees centigrade	-
FiO_2_	70	%	-

**Table 2:** Blood tests taken on admission GFR, glomerular filtration rate; NT, N-terminal; PTR, prothrombin ratio; INR, international normalized ratio; APTT, activated partial thromboplastin time. Abnormal results are identified in bold.

Parameter	Result	Unit	Normal Range
Sodium	139	mmol/L	133-146
Potassium	4.1	mmol/L	3.5-5.3
Urea Serum	6.2	mmol/L	2.5-7.8
Creatinine	119	µmol/L	59-104
Estimated GFR	54	mL/min	>90
Total Bilirubin	25	µmol/L	5-21
Alanine Aminotransferase	37	U/L	10-41
Alkaline Phosphatase	83	U/L	30-130
Adjusted Calcium Serum	2.38	mmol/L	2.2-2.6
Inorganic Phosphate Serum	2.40	mmol/L	0.7-1.4
C-Reactive Protein	36	mg/L	<5
Creatinine Kinase	387	U/L	40-320
Magnesium	0.74	mmol/L	0.85-1.1
Troponin T Serum	18	ng/L	<14
NT- pro-B-type natriuretic peptide	294	ng/L	<125
PTR/INR	0.98	-	
APTT	23.8	Seconds	24-37
Haemoglobin	143	g/L	130-180
Platelets	230	×10^9^/L	140-400
White Blood Count	13.2	×10^9^/L	3.6-11
Neutrophil	12.5	×10^9^/L	1.8-7.5
Lymphocytes	0.3	×10^9^/L	1.0-4.0

**Figure 3:**
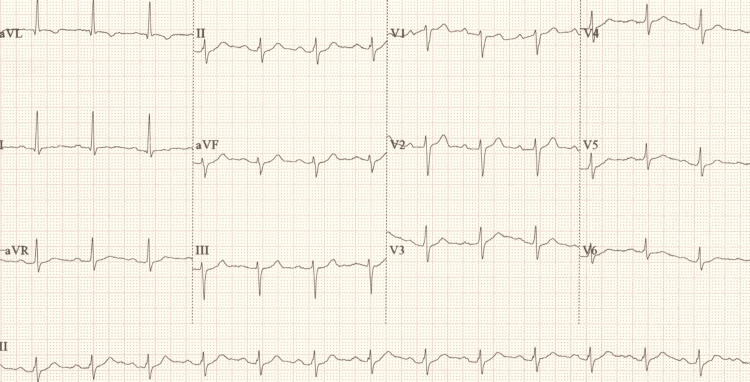
ECG taken after cardiac arrest demonstrating similar findings compared to admission ECG ECG, electrocardiogram.

**Figure 4:**
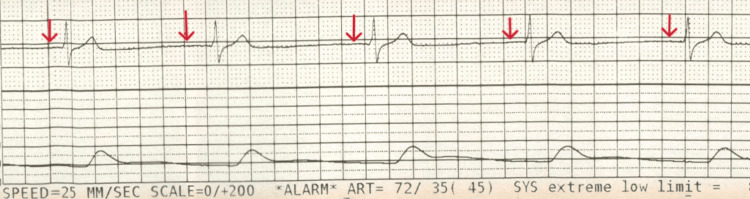
ECG rhythm strip taken at the time of symptoms and haemodynamic instability showing a junctional rhythm due to the lack of P waves before QRS complexes (marked with red arrows), with a ventricular rate of 49/min ECG, electrocardiogram.

In the context of the above investigations and results, he was diagnosed with type 1 respiratory failure secondary to inhalation pneumonitis and was started on non-invasive ventilation (NIV) (CPAP) at a fraction of inspired oxygen (FiO_2_) of 0.7. He was admitted to the intensive care unit (ICU) for close monitoring given his co-morbidities. Within 4 hours of admission to the ICU, he experienced a 10-15-second episode of syncope associated with cardiac standstill, which resolved spontaneously. This was soon followed by a witnessed period of bradycardia leading to asystole. One cycle of CPR was performed and resulted in return of spontaneous circulation (ROSC). The post-ROSC ECG showed borderline first-degree heart block with a PR interval of 214 ms (Figure 3). He continued to experience transient episodes of symptomatic bradycardia, with real-time rhythm strips (Figure 4) confirming a junctional bradycardia correlating with pre-syncopal symptoms and hypotension. His echocardiogram showed a normal ejection fraction of 64% with no regional wall motion abnormalities. Differentials here included electrolyte abnormalities, structural cardiac disease and medication side effects; however, these were excluded given normal electrolyte blood markers, a structurally normal echocardiogram, and the patient not being on any regular medication likely to predispose bradycardia. Subsequently, a discussion with the national toxicology consultant on call identified dichloromethane and toluene - both components of POR-15 - as potential cardiotoxic agents, but there was no specific toxicological management, and conservative management was advised. Given his ongoing junctional bradycardia with haemodynamic instability, the local cardiology team was consulted, and he was commenced on an isoprenaline infusion due to the frequent episodes of junctional bradycardia and loss of cardiac output. Transcutaneous pacing pads were attached to be used on standby. The patient’s heart rate and blood pressure demonstrated improvement following administration of isoprenaline, with heart rate increasing to the 60s and systolic blood pressure maintained above 100 mmHg. After consultation with the regional cardiac centre, he was transferred for close cardiac monitoring and consideration of temporary intravenous pacing in view of the high risk of recurrence. 

He made a full recovery without requiring transvenous temporary pacing and was subsequently discharged from the hospital eight days after admission to the cardiac centre. Given the complete resolution of symptoms and no recurrence during admission, no further cardiology follow-up was arranged by the tertiary centre or us. It was felt that this was likely a one-off exposure with complete resolution. The pneumonitis features on his initial chest x-ray had resolved when he was seen in the respiratory clinic follow-up three months post-discharge.

## Discussion

In the acute setting, diagnosing inhalation-induced arrhythmias can be very challenging as the laboratory tests are limited by their cost and availability and the clinical and pathological findings are often non-specific. Therefore, diagnosis is often made depending on relevant clinical findings, a high suspicion of the index and evidence of multi-organ involvement. This case also highlights the importance of detailed history taking. Unfortunately, our case lacked definite laboratory tests for the diagnosis of toluene intoxication due to the limited diagnostic facilities available at the local hospital. This was predominantly a clinical diagnosis, which was established by a compatible clinical presentation and ruling out likely other possible causes. 

Here, the patient developed severe symptomatic junctional bradycardia, which occurred in the absence of known cardiac disease. The cause was attributed primarily to the hydrocarbon components of the POR-15 solution [5,6]. The underlying mechanism involves increased myocardial sensitivity to adrenaline, contributed by the inhibition of calcium influx and altered sodium and potassium channels' activity due to the toluene molecule [7,8]. Furthermore, experimental studies have shown that toluene can lead to cell apoptosis [9]. Current European Society of Cardiology (ESC) guidelines advocate for a conservative approach to the treatment and correction of reversible factors prior to consideration of pacing and highlight the use of prolonged ambulatory ECG monitoring for identification of arrhythmia and appropriate correlation with symptoms [10]. However, currently, there are no specific antidotes for toluene toxicity. Isoprenaline was used as opposed to atropine as it directly increases junctional automaticity and conduction, whereas atropine mainly acts on the sino-atrial node.

## Conclusions

This case illustrates a rare presentation of symptomatic junctional bradycardia from chemical inhalation, emphasizing the need to consider toxin-induced causes when supported by clinical and investigative findings. Identifying the causal chemical agent, pursuing reversal where possible, and adopting conservative management are key, as such cases are often self-limiting. It also underscores the importance of timely rhythm monitoring during symptoms to guide management, as well as early involvement of specialist teams for appropriate advanced care. 
